# The Fourth Session of the Research Society of the American Red Cross in France

**Published:** 1918-04

**Authors:** 


### The Medical Bulletin

N° 6 PARIS APRIL 1918

### RESEARCH SOCIETY REPORTS

### THE FOURTH SESSION OF THE RESEARCH SOCIETY OF THE AMERICAN RED CROSS IN FRANCE

March 15 and 16, 1918, at the Institut Pasteur, Paris.

At the first meeting of the Session General CUTHBERT C. WALLACE, R. A. M. C., presided. The subject was Traumatic Shock.

Major GEORGE W. CRILE, M. R. C., presented a paper in which he declared that the outstanding fact in shock is loss of the power of transforming potential into kinetic energy, the loss of power to produce heat and motion, the diminution of the internal respiration of the body — including a diminution of oxidation, and therefore a reduction of the working power of the brain. The working powers of the glands and muscles is also reduced. The heart muscles are affected in this manner as well as the voluntary muscles. The respiratory center of the brain, however, shows no such diminution of power, for its activity is increased.

The appearance of a patient suffering from shock is that of a state of prostration. The face is white and shrunken. Saliva and the gastric and internal juices are reduced. There is little excretion of urine. The mind is dulled, and the patient may be restless or in a stupor. The body temperature is sub-normal; the circulation is feeble; and the blood is sequestered and pooled in various parts of the vascular system.

Interference with intracellular oxidation causing shock may result from a chest wound which interferes with lung ventilation, or from want of food, water, and sleep. Such interference produces intracellular acidosis. In this respect, regular sleep is as essential as oxygen and food. Anxiety during battle increases the transformation of potential into kinetic energy and causes a corresponding increase in the acid by-products. If these by-products are produced faster than they are eliminated, a state of acute acidosis is established which interferes with oxidation and hence produces prostration and shock.

Loss of blood causes further interference with internal respiration, because it diminishes the supply of food to the cells and reduces the elimination of waste products from the cells, thus interfering with oxidation.

Diminution of oxidation, followed by intracellular acidosis, prostration, and shock, may result from any wound which cuts off the air, food, or water supply, or from phosgene gas, CO gas, or loss of sleep. The inhalation of anesthetics and rough surgical operations may produce the same effects.

One of the most potent causes of shock is the goading of a compound fracture in transport from the front. The state of exhaustion which follows the strain and exposure of battle predisposes a patient to shock from minor causes. Hence every effort should be made to induce sleep and to provide comfortable means of transportation. Stretcher-bearers should be impressed with the necessity of using the utmost precaution in handling compound fractures. The Thomas splint is one of the most effective means of preventing shock that this war has developed.

If there is active bleeding, it should be arrested promptly. An exsanguinated man or one with a condition of shock should be hurried to a hospital with a resuscitation team on duty. He should have blankets under him as well as over him, and should be given hot drinks — tea, coffee, cocoa — *well sweetened*. If there is a sucking hole in the chest, it should be closed at once, preferably by suture. For intense pain without definite cyanosis, morphia in moderate and, if necessary, repeated doses may be administered. Maximum doses should never be given.

A resuscitation team should include a surgeon, a nurse, and an orderly. They should be occupied exclusively with the treatment of shock, either before or after operation. The ward devoted to this work should be equipped with means for giving warmth, comfort, and rest, and it should be provided with apparatus for administering intravenous infusions and blood transfusion, and for the arrest of hemorrhage. There should be a simple means of keeping records.

If a patient does not respond to the simple physiological means of restoration, which are the most natural and therefore the most effective, and if he continues to decline, prompt radical treatment is indicated. Blood transfusion has been found to be the most potent means of overcoming profound shock. A 5 o/o solution of sodium bicarbonate and glucose is an excellent substitute, especially if there is gas infection. If the response from this method of treatment is not satisfactory, blood transfusion should be performed. Bayliss' solution holds up better than normal saline and perhaps better than hypertonic solutions. The speaker declared that in the experience of the Lakeside Unit, substitutes for blood have not given such good results as blood transfusion.

Results from blood transfusion are best when the duration of the shock is brief, and when hemorrhage is a principal factor. When the state of shock is so profound as to have occasioned intracellular changes, especially in the brain, the prognosis is bad, and all treatment liable to failure. One should at least avoid delay and, in case of doubt, practise transfusion.

*Morphine.* — If there is no cyanosis, morphine is one of the best agents in the treatment of shock. It should be administered not merely to relieve pain, but, as in acute peritonitis, for deep narcotization. This treatment should not be given alone, however, but in combination with rest, water, warmth, inclining of the bed, and blood transfusion. Cyanosis which is apparent in the gray blue patient, is a contra-indication for morphine, because morphine interferes with the mechanism which overcomes cyanosis. The special value of morphine is in producing deep, untroubled sleep, the best intracellular restorer. Nothing but oncoming gas gangrene or acute hemorrhage justifies rousing a shocked patient from normal sleep.

*Surgical Operation in Shock.* Anesthesia should be local, regional, or sacral, with novocain or with nitrous oxide. The operation should be as light and brief as possible.

*Chest Cases.* Positive pressure gas and oxygen apparatus inflates the lungs, overcomes cyanosis, and maintains anesthesia. With a high pressure nitrous oxide oxygen apparatus, anesthesia and lung ventilation can be maintained even when both sides of the thorax are widely and simultaneously opened. All pulling on the lung should be avoided by adequate incision. Blood transfusion helps tissue ventilation as well as circulation.

*Abdomen.* After blood transfusion novocain may be applied locally, and analgesia produced by nitrous oxide: nitrous oxide should be supplemented with ether during the exploration. The operation may be completed under analgesia again. Acidosis, attending all forms of deep inhalation, may in this manner be avoided.

*Shock and Phosgene Gas.* Since phosgene gas and shock interfere with internal respiration, causing acidosis and interfering with oxidation, it is important that further acidosis such as results from deep breathing anesthesia and from operative trauma be avoided. Local, regional, or low spinal anesthesia with a minimum of novocain, supplemented with analgesia should therefore be the chosen methods of anesthesia. At the close of the operation, the administration of oxygen under pressure has a distinct value.

*Shock and Gas Gangrene.* In cases of gas gangrene treatment by intravenous injections of sodium bicarbonate is at its best. Since gas gangrene itself causes deep acidosis, it is again important to avoid inhalation anesthesia. Intracellular lesions of gas gangrene, especially in the liver and brain, are similar to those produced by prolonged ether or chloroform anesthesia. Vomiting in gas infection is probably due to the same cause as vomiting in anesthesia and other forms of acute acidosis.

*The Brain.* Local anesthesia is an advantage even in operating for brain wounds, in which the destruction is principally mechanical, and hence less likely to interfere with intracellular respiration.

*Post-operative Care.* The general principles and practices of post-operative care are the same as those for pre-operative treatment. The routine practice of administering rectally by the Murphy drip a 5 o/o sodium bicarbonate and 5 o/o glucose solution has given advantageous results in thousands of cases.

*Advantages and Limitations of Intravenous Infusion.* Normal saline infusion — intravenous or subcutaneous — are useful in cases of moderate exhaustion. It is found experimentally, however, that in animals normal saline solution when infused intravenously, even in excessive amounts, does not raise the normal blood pressure, because it runs out of the circulation through the normal channels as rapidly as it enters. Water is of most value when absorbed through the alimentary canal.

*Hypertonic solution of sodium bicarbonate or of sodium carbonate*, both experimentally and clinically, is found to be more potent than normal saline in raising and sustaining the blood pressure and in relieving exhaustion. Its effects, however, are much weaker and much less enduring than those produced by blood transfusion. It delays but does not prevent the production of shock.

*Bayliss' solution of gum* raises the blood pressure and sustains it well, but the final results from its use are little better than those from sodium bicarbonate solution.

*Magnesium salts* give promise of effective aid in overcoming shock, for they seem to aid in intracellular restoration when injected intravenously. A " magnesium sleep " lasting about two hours can be produced and a diminution of the edema in the liver caused by exhaustion takes place much as in normal sleep.

Taken alone, however, magnesium salts are cardiac depressants and their value in shock cases, now under observation, is not yet established.

*Morphine.* Laboratory research has confirmed clinical experience in demonstrating that morphine diminishes shock, protects against pyogenic infections, and helps in many ways to sustain life during a critical condition. When the patient is cyanosed, however, it is certain that morphine interferes with the natural means of throwing off the cyanosed condition. During the acute cyanosed stage of shock — no morphine.

The speaker concluded with the following list of " dont's ":

Don't annoy, delay, nag, and further injure by taking off shoes or clothing in the front area.

Don't forget to throw Thomas splints as far forward as the soldier goes.

Don't forget that there is a man as well as a wound; the man may be killed while the wound is being saved.

Don't forget that the bottom of the stretcher needs blankets more than the top of the man.

Don't forget the value of reassurance.

Don't give stimulants — the patient is not in need of stimulants — for the battle is a stimulant; the whistling and bursting shell is a stimulant; the shattered bone is a stimulant; in other words,

Don't give the soldier an overdose of the thing which is killing him.

In doubt, don't delay, but give blood transfusion. By the time death is 100 o/o assured, failure of treatment is 100 o/o assured.

Don't do a 10 o/o mortality anatomical operation when there remains only a 5 o/o physiological chance of life.

Don't fail so to organize and equip and instruct each sector of the army from regimental surgeon to the base hospital that an approximate uniformity of conception and practice may be established.

Don't establish a ward for the survival of the fittest, but strive to make every one fit to survive.

Lieutenant Alexander L. Prince, M. R. C., read a paper entitled " Observations on Shock Acidosis ".

Noteworthy among the theories as to the cause of shock, he remarked, was that of Henderson, known as the Acapnia theory. According to this theory, as first stated in 1910, the primary factor in the shock cycle is a reduction of the carbon dioxide contents of the blood (acapnia) resulting from the hyperpnea induced by severe sensory stimulation (pain hyperpnea). The reduction of carbon dioxide in the blood causes either fatal apnea or a failure of the circulation through cardiac tetanus. The failure of a hypothetical veno-pressor mechanism, resulting in a diminished return flow to the heart, is also considered as a possible cause of the circulatory failure. Early in his investigations Henderson suggested that acidosis from tissue asphyxia would likely follow the progressive failure of the circulation.

As a result of recent investigations in Henderson's laboratory, the original theory has been modified concerning the exact relation of acapnia to acidosis. The acidosis instead of being considered a direct result of a failing circulation is given the nature of a compensatory process. The hyperpnea resulting from prolonged sensory stimulation leads to a fall in the carbon dioxide contents of the blood. The partial removal of the normal respiratory stimulant (carbon dioxide) would result in fatal apnea, were it not compensated by a corresponding production or retention of acid in the body fluids, so that the hydrogen ion concentration of the blood is maintained at a level permitting respiration to go on uninterrupted.

In experiments on dogs, using solid anesthetics which do not produce acidosis as is the case with ether and chloroform, shock was induced by exposing and manipulating the intestines. Samples of blood were withdrawn at regular intervals throughout the experiments and the blood analyzed for its carbon dioxide contents, that is to say, the actual percentage of carbon dioxide found in the blood immediately after its removal from the animal. The blood was also tested for its carbon dioxide capacity, in other words, its ability to take up carbon dioxide when balanced with a sample of alveolar air of normal tension (5.5 o/o).

In all instances the shock procedures were followed by a progressive fall in the carbon dioxide contents and capacity of the blood, indicating a progressive acidosis. The alveolar air of the animals was also analyzed and showed a fall of carbon dioxide tension parallel to the acidosis curve. The blood pressure, likewise, fell progressively. The results of these experiments have been recently confirmed by the observations of Cannon on man.

The compensatory character of "shock acidosis" receives support from the converse experiments in which the animals were made to rebreathe their expired air so as to raise the tension of the alveolar carbon dioxide. The blood of these animals showed an increased capacity for carbon dioxide, when balanced with a sample of alveolar air of normal carbon dioxide tension. Thus, the organism attempts to compensate for the increased hydrogen ion concentration of the blood, induced by rebreathing, by a reduction of the fixed acid or an increase of the alkali in the blood. It would seem, therefore, that carbon dioxide controls the reaction of the blood and that in our shock experiments, acapnia is the cause of the acidosis, rather than its effect.

The acapnia shock theory as stated at present necessitates a period of hyperpnea of sufficient duration to initiate the acidosis. In other words, the alveolar carbon dioxide must remain at a low level long enough to initiate a compensatory increase in the acidity of the blood. Cannon and his collaborators have reported a large series of shock cases in all of which hyperpnea was not apparent. As the respiratory volume per unit time was not determined, the possibility of pulmonary overventilation still remains. Experiments on man, from Haldane's laboratory, show that pain even of a moderate character is associated with a fall in the percentage of the alveolar carbon dioxide.

From the facts now at hand, it is evident that additional study will be required to establish the exact position occupied by acidosis in the shock cycle. Whatever this relation may be, we have in acidosis a condition which, as shown by Cannon, forms a link in a vicious circle which tends to increase the degree of acidosis existing. While this vicious circle may be broken and the blood restored to its normal reaction by means of alkaline therapy, the experimental evidence at hand gives us additional means through which we may more completely restore physiological equilibrium. In shock acidosis the internal environment of the organism is not only toxic through the presence of an abnormal amount of fixed acids, but the decreased amount of carbon dioxide in the body fluids is also an abnormal state which should be remedied. It should be kept in mind that the normal environment of the body cells is a fluid containing 45 to 50 volumes per cent of carbon dioxide. It is a notable fact that carbon dioxide is not merely an excretory product but that it represents one of the normal constituents of the internal environment of the organism. For instance, while the hydrogen ion concentration of the blood affects the rate and depth of respiration, the experiments of Hooker and Wilson prove conclusively that carbon dioxide is the specific stimulant of normal respiration. In the perfusion of the excised heart the speaker and his colleagues have always found that oxygenated blood mixtures poor in carbon dioxide prevent the complete relaxation of the ventricles so that with a given filling pressure the systolic discharge is very much decreased, and the rate of circulation consequently impaired. As shown by Henderson and others normal intestinal peristalsis is dependent upon the presence of carbon dioxide in quantities such as are found in body fluid. In addition to the fact that carbon dioxide is essential to many of the activities of the organism, the experimental evidence that this gas regulates the reaction of the blood furnishes a therapeutic indication for the treatment and prophylaxis of shock acidosis which at least deserves consideration and study. The beneficial results obtained by Porter in the treatment of shock by carbon dioxide inhalations gives this view further support. While alkaline therapy is surely indicated in shock acidosis the inhalation of 5.5 o/o carbon dioxide in addition may prove a valuable procedure. For that purpose, the speaker and his colleagues have devised a simple rebreathing apparatus permitting the maintenance of the alveolar carbon dioxide at a normal level in shocked individuals. As soon as material is available they hope to communicate the results obtained by this combined method of treatment

Major Cannon, M. R. C., stated that the work which he had to report[1] was carried on through four months last summer in the First British Army, under the favoring interest and counsel of General Cuthbert Wallace, by Capt. John Fraser, Capt. E. M. Cowell, Capt. A. N. Hooper, and himself. The first observations which led Major Cannon and his colleagues to look for facts instead of following theories were made by General Wallace and other surgeons cooperating with him. They found no evidence of accumulation of blood in the abdominal vessels of shocked men, as was commonly assumed. This observation was subversive not only of current theories on shock but of recent suggestions for treatment.

It seemed desirable therefore to sit down before shock cases and learn what they could about them. One of their first activities was to examine the blood, not only from the capillaries but also from the veins. Capillary blood was taken from ears, finger tips, toes, and the mucous membrane from the interior of the mouth. In all cases of shock it was found that the capillary blood count was higher than the venous count; not infrequently the difference was as great as 2,000,000 or 2,500,000 corpuscles per cubic millimeter. From this indication, therefore, the " lost blood " of shock was to be found in congestion or concentration of the blood in capillary areas.

Another phenomenon which was observed through the cooperation of Capt. Cowell in the front lines and of others working further back was the bad effect of cold on wounded men. These men would leave the front lines in fair condition and on becoming cold

1. For a full account of these observations see Pamphlet No. II of the Shock Committee of the British Research Society. See also p. 463 of the present number.

as they were carried back they showed signs of shock. Cold was known to produce capillary stagnation of the blood and it was probable that cold operated to increase the segregation of blood in the capillary regions.

Observations were also made on the chemical condition of the blood in these cases. It was found that in men suffering from shock, hemorrhage, or gas infection, a condition of acidosis was present as indicated by a diminished amount of sodium bicarbonate in the blood. The degree of acidosis in shocked men corresponded roughly to the degree of lowering of the blood pressure.

It had been noted that during a surgical operation there was a change of the blood in normal individuals in the direction of acidosis. Major Cannon and his colleagues found that this change occurred also in shocked men but with an important attendant alteration of blood pressure if acidosis was already present or if the alkaline reserve was reduced to such a degree by operation that acidosis was established. Thus, though there might be no noteworthy fall of blood pressure accompanying the lowering of the alkaline reserve above the acidosis level, the average change in the blood pressure in shocked men, when the acidosis was increased by operation, was from 88 mm. Hg systolic, 62 diastolic, to 62 systolic, 31 diastolic. The acidosis might be changed from a mild to a marked degree. Both the sharp fall in the alkaline reserve and the marked drop in the blood pressure might occur in less than fifty minutes. It was clear from this evidence that the shocked man was liable to two serious alterations in his condition as a consequence of operative interference.

The correspondence between the degree of low blood pressure and the degree of acidosis in shock, as well as the correspondence between the fall of blood pressure and fall of the alkaline reserve during operation, indicated that there was a causal relation between the two conditions. It was certain that low blood pressure could produce an acidosis through inadequate oxygen supply to the tissues. It seemed possible that there was a relation in the other direction, i. e., that the acidosis might produce low blood pressure. This possibility was tested in experiments carried on in cooperation with Prof. Bayliss. If an animal was anesthetized with urethane and the alkaline reserve was reduced by injecting acid intravenously at a slow rate so that the heart and the bulbar centers were not injured, the blood pressure fell after the injection ceased until the shock level was reached and might remain there until death supervened. The same was true for animals anesthetized with chloralose. The speaker did not wish to claim too much for these observations because in similar experiments by Dale and Richards, performed on animals under ether, the phenomenon did not occur. The explanation of the difference was not clear. For the present the speaker wished only to point out that under the circumstances of the experiments an induced acidosis equivalent to that in human cases caused a condition of shock. If that could be done by injecting acid from the outside, might it not be possible to obtain the same effect by acid products originating in the body?

The first experiment in this direction was done by clamping the aorta above the iliacs; the blood supply to the hind limbs was thus stopped. This anemia of the tissues was continued for an hour before the clamp was removed and three minutes afterwards blood samples were taken. The difference between the two showed that the acid swept from the anemic tissues into the general circulation was sufficient to bring about a state of acidosis. And this acidosis was accompanied by a condition of shock.

This observation was related to the work of Fletcher on the development of lactic acid in muscle deprived of its oxygen supply. A muscle deprived of oxygen would produce 0.5 o/o of its weight of lactic acid. Fletcher also noted that if muscles were injured, lactic acid production proceeded in the absence of oxygen much more rapidly than if they were not injured. In the experiments the speaker and his colleagues tried injury of the flexor muscles of the thigh and found that subsequent to the injury there was a gradual increase of acidosis and when this had reached what seemed to be a critical point the blood pressure began to go down and continued falling until the animal was brought into a condition of shock. In all probability there were other substances besides the acid given out by the injured muscle, which might cause vasodilation.

These observations suggested the possibility that there might be in cases of secondary shock, i. e., shock appearing in wounded men some time after the injury, a primary acidosis, due to injured tissues, which would lead to shock. If it were true that acidosis or a production of acid metabolites led to vasodilation, then the low blood pressure of shock and of hemorrhage by inducing acidosis would make worse the existent condition; also the low blood pressure in cases of gas infection might be accounted for as due to acid given off from dying and disintegrated muscle.

One started without a theory, but it was difficult to avoid ending with one. There was good evidence that the acid metabolites given off by an active organ caused local vasodilation attended by a passage of lymph from the blood vessels into the tissues of the organ. Might we not reasonably regard secondary shock as due to the diffusion of acid metabolites so generally through the body that this process of vasodilation and escape of lymph became pathological and resulted in such diminution of blood volume that the heart would not have an adequate supply to work upon and the low blood pressure of shock would secondarily follow?

There were a number of practical suggestions which arose from the foregoing considerations. In the first place, since cold led to capillary stagnation it was a matter of primary importance that the wounded man should not be allowed to become cold. Every effort should be made to conserve his body heat, which tended to escape because he sweated; external heat should be applied by placing hot water bottles about him while he was being carried, and warming him with electric covering when he reached his hospital bed.

It should be recognized that when a tourniquet was removed there was swept from the injured and anemic tissues into the general circulation substances which were liable to be deleterious, as was shown in the experiments previously mentioned.

Again, since the circulating acid in the body was probably lactic and was capable of being oxidized, it was important that nothing be done to diminish the oxygen supply. The giving of doses of morphia so large as to slow the respiration would lead to an acidosis by itself. There was the possibility, therefore, that an over dosage of morphia would increase an existent acidosis. Also during operation there should be avoidance of rebreathing expired air from which some of the oxygen had already been removed, for in this way the oxygen supply of the body was reduced.

It was possible to avoid the sharp increase of acidosis and the marked drop of blood pressure during operation by an intravenous injection of warmed 4 o/o sodium bicarbonate at the beginning of operation. The result was that at the end of operation the alkali was normal and the blood pressure was even higher than at the beginning. Recently the British Medical Research Committee had arranged for supplying sodium bicarbonate (2 o/o) in a gum acacia solution (6 o/o) — a combination which would doubtless remain in the blood vessels longer than the simple salt solution and which would at the same time raise the blood pressure and neutralize the acidosis.

The evidence that operation had a serious effect on men with a low alkaline reserve indicated that it was desirable to provide alkali for shocked men, and consequently the speaker and his colleagues had advocated the giving of alkaline drinks at advanced posts in the Army Medical Service.

There was clear evidence that if a low blood pressure were allowed to continue, changes were brought about, not only in the blood vessels, but also in their nervous control, which were difficult to reverse. A low blood pressure which could be easily recovered from if treated early could not be recovered from if it was allowed to proceed for some time. This fact emphasized the desirability of obtaining treatment for these cases as early as possible after the shock appeared — a point which deserved the greatest emphasis.

Major Rist presented a paper by Dr. Ch. Vincent on shock resulting from war wounds. Dr. Vincent wrote as a regimental doctor and neurologist who had seen his patients on the spot where they fell wounded, at the first aid post, and, in many cases, at the ambulance where they were treated. Though he had been present at some of the hottest battles of the war, he had, unlike the surgeons, seen comparatively few cases of immediate or early shock, and he explained this difference in experience by the fact that surgeons at an ambulance often diagnosed as " shock " a condition which was due to hemorrhage: having seen no blood, they concluded there had been none, or at all events very little. The writer, however, had frequently seen the same patients lying in pools of blood, though the hemorrhage had subsequently ceased in some cases — even without the application of a garrot. The big arteries were not the only source of extensive bleeding; men suffering from bone shattering with muscle injury sometimes lost an incredible quantity of blood, and, *in the majority of cases*, when the surgeon had diagnosed " shock ", hemorrhage was the real cause of the condition. The cases in question consisted of hemorrhages of the limbs, of the abdomen, and of the thorax.

Is hemorrhage in itself a necessary and sufficient cause of shock? In other words, can the trauma alone, unaccompanied by hemorrhage, bring about a state of shock? Or, if hemorrhage is a necessary agent in producing shock, is it not powerfully aided in certain cases by other causes?

In the writer's opinion no other cause is so frequent or so evident as hemorrhage. It would seem that extensive fractures, abdominal injuries, multiple wounds, and certain special injuries of the spinal cord, though accompanied by comparatively slight loss of blood, may give rise to a condition identical with or analogous to that of shock. It is impossible to pronounce definitely on the question at the present time owing to the difficulty of observation and of carrying out autopsies in a satisfactory manner. If, however, it is difficult to be certain as to the direct or reflex action of cardio- or vaso-motor phenomena, one may, perhaps, be more precise as to certain factors invoked as causes of shock. Cerebral commotion, agitation, chill, exhaustion, cannot, of themselves, produce shock. They are manifested by syndromes peculiar to each which are very different from that of shock.

The fighting man consumes his energy with such rapidity that he is peculiarly susceptible to hemorrhage or other physical disturbances. During a battle the blood pressure of the soldier is generally below the normal; in 50 per cent the maximum is from 105 to 110 and the minimum from 60 to 70. Moreover, experience has proved that on the battlefield quite another cause — namely, a common infection — can lower the arterial pressure to an extraordinary extent.

It is beyond question that certain men, during battle, are predisposed to shock. Even when victorious, exhaustion renders them susceptible to it; and how much more so must they be when the army loses ground and the wounded pass hours in shell holes, cold, with nothing to drink, and exposed to barrage fire. To loss of blood must be added pain, chill and exhausting anxiety as accessory causes. Defeat and mud are powerful factors in shock.

*Treatment of shock on the field.* The best prophylactic treatment of shock is :

1. To hold the battle field, i. e. to be stronger than the enemy and to have artillery which silences his.

2. To bring in the wounded speedily : prisoners should be employed for the purpose as the regimental medical service is insufficient.

3. To have a well organized regimental service. Each stretcher-bearer, each officer, each man, if possible, should know how to fix a garrot. The use of the garrot has been much criticized, but if it causes the loss of a limb it may save a life. Many men die unnecessarily from hemorrhage on the battle field and at the ambulance.

4. A more general use should be made, even at the first aid post, of morphine, camphorated oil, ether, and serum.

Captain E. M. Cowell, R. A. M. C., spoke on the early stages of wound shock. He stated that his post in the front lines had enabled him to observe the physiological condition of the fighting soldier both before and after injury. In periods of calm the soldier's blood pressure is normal. Under the excitement of shell fire and bombing, however, the blood pressure is raised. The fatigue of trench life induces a tendency towards concentration of plasma, sluggish peripheral circulation, and accumulation of waste products of muscular metabolism. The urine of men under such conditions is dark, scanty, and loaded with phosphates. In times of excitement the systolic blood pressure may range from 140 to 160 mm. and the diastolic, from 70 to 100 mm. In the heat of actual battle, no doubt these conditions are all present to an increased extent.

Three classes of wounds are to be considered with reference to the incidence of wound shock: trivial wounds, moderately severe wounds, and serious wounds.

Trivial wounds, such as scratches on the scalp and other superficial injuries, are often accompanied by psychical disturbances, pallor, and sweating. Generally in such cases a normal blood pressure is soon restored, although in "highly strung" patients, a period of excitement may follow with a systolic pressure in some instances as high as 180 mm. Rest quickly restores the pressure to normal.

In moderately severe wounds — those that involve considerable destruction of tissue, but that do not immediately endanger life — primary wound shock does not as a rule occur. If examined immediately after injury, such cases often show no sign of shock or of general systemic disturbance. Blood pressure may remain within normal limits. After the lapse of a short time such cases may develop secondary shock as a result of factors which may often be avoided.

Serious wounds — those presenting a dangerous surgical condition — are likely to develop an early condition of shock, or what is genuine primary shock. Death usually occurs before such cases can get very far back.

In cases of secondary shock, if the man is properly looked after, the prognosis is not usually serious. If, however, conditions are unfavorable, a gradual depression may continue and death occur in 8 or 10 hours. The speaker cited the case of a man whose pressure immediately after injury was normal. He was exposed to cold and shell fire for some time after injury, and arrived at the first aid station in a pulseless condition. By the time he reached the clearing station he was in a state of profound shock. He was warmed and within half an hour his pulse returned. Another man, with a simple bullet wound through the thigh, arrived in a state of shock. He had lost no blood, and was simply cold. He was given hot water and was well wrapped up. He was soon restored.

Primary wound shock occurs generally when the wound of necessity must prove fatal. The symptoms result immediately, and hypertension is observed from the earliest moments. It is produced more readily in men of temperamental instability. After a few hours, unless treatment leading to recovery is possible, primary wound shock merges into secondary wound shock under the influences of cold, pain, and anxiety. In light cases the symptoms of primary shock pass away quickly, and recovery is rapid.

Colonel Sir Almroth Wright, R. A. M. C., stated that we owed the knowledge that acidosis is an element in the condition of surgical shock, to Majors Crile and Cannon. This discovery was of special value in drawing attention to an element in the vital machinery of the body, heretofore imperfectly explored — the means by which alkalinity is maintained in spite of the large output of acid waste products from the body.

In attempts to explain the cause of acidosis in the wounded man, experiments on rabbits, anesthetized with ether, were carried out. Arteries were clamped and later released. It was observed that a considerable quantity of non-expirable acid was in this manner conveyed into the blood stream, due to the resumption of the metabolic exchanges which had been suspended during the collapse of circulation. Such an effect might be produced by ligature, tight bandaging, or the lowering of arterial pressure. Similar results, even more pronounced, were obtained by the immersion of the animals in an ice-bath.

The restoration of the circulation was seen to increase the acidemia, and it was observed that blood returning from the muscles was more acidosed than blood taken from the right heart or *vena portae*. The normal alkaline reaction in the muscle was gradually restored, and the acid remaining in the blood was slowly burned off in the system.

That hemorrhage may be a cause of acidosis was experimentally proved by Milroy. This was due not only to the lowering of arterial pressure, but also to the removal from the blood stream of sodium and potassium carbonates — the alkali available for neutralizing the acid.

Experiments were carried out in which metabolism was greatly increased by faradization. Acid in the muscle after such tetanization was found to be the same as that resulting from cold immersion. Acidemia from tetanization, like that from cold immersion and arterial occlusion, passed off in a few hours.

All of these factors might enter into the condition of the wounded man. In the light of Fletcher's work on the metabolism of muscle with relation to oxygen, it appeared to the speaker that we were dealing with an acidemia produced by lactic acid evolved in muscles by an impairment of their oxygen supply, similar to that which develops in a dead body.

The speaker believed that acidosis of a similar derivation was probably associated with chilblains, trench foot, and certain bacterial infections, such as gas gangrene and Asiatic cholera.

Acidosis in chilblain and trench foot was like that resulting from anesthesia, due in the main to the shutting down of circulation. It was highly desirable, as Cannon had insisted, not to add to the acidosis of shock, the further acidosis of anesthesia.

As the speaker had shown in 1917[1], gas gangrene was characterized by an extreme acidosis. It was at present clear that this acidosis was due not merely to the acid produced by the organism, but also to acid elaborated by muscle, which tended to create a favorable medium for the spread of the organism. Thus it was found that a fulminating gas gangrene could be produced in the dead animal by the passage of the acid into the blood after death. It was therefore probable that shock predisposed a patient to the development of gas infection. Experiments on animals showed that the Welch bacillus sown in blood drawn after tetanization or cold immersion, could be recovered in cultures with gas formation, whereas the bacillus sown in blood from the normal animal developed in no such manner. At the height of the toxemia, in gas gangrene also, the circulation tended to break down, and thus acidosis became still more serious.

In Asiatic cholera, as Rogers had shown, acidosis played an important part. Here also, it was probably due to the impairment of the circulation in the algid stage of the disease, when the blood had been inspissated in consequence of the alvine discharges.

There was an obvious bearing of the points so far made upon the practical treatment of shock — the warming of the patient and the intravenous injection of alkali, as recommended by Cannon.

The speaker believed, that although warming was an excellent means of prophylaxis against the condition of shock, it was likely to lead to serious consequences if applied to a patient in a fully developed condition of shock. The precipitate washing of muscle acid into the blood might be perilous to life, because it conveyed additional acid into the blood stream thus increasing the acidemia. To avoid such resuscitation acidemias, it was best, the speaker believed, to commence procedures by a bicarbonate of soda injection. It was highly important, also, to avoid anesthesia acidosis, by delaying operation, in such cases, until the blood was

1. WRIGHT, *Lancet*, Jan. 6, 1917 and WRIGHT and FLEMING, *Lancet*, Feb. 9, 1918.

shown by a test to have returned to a normal alkalinity. Direct titration in capillary tubes made it possible to test a sample of serum in two or three minutes.

Dr. J. L. Roux-Berger read a paper on the etiology and pathology of shock. He stated that he had had the great advantage of studying this question from two points of view, i. e., in an advanced post under extreme pressure and in a surgical station 15 kilometers from the lines at a time of average activity.

Two categories had been established: primary shock and secondary shock, the distinction between the two being based on the rapidity of the sequence of the phases. Such a distinction hardly seemed justified, however, as apart from "cerebral commotion" (which should not be considered as shock) and certain conditions seen in abdominal cases, primary shock was very rare.

As regards the pathogenesis, there was a tendency to establish the following classifications:

Nervous shock
Infectious shock
Hemorrhagic shock
Septic shock.

1. *Nervous shock.* The English "shell shock," he declared, corresponded to what the French called "cerebral commotion." Nerve specialists regarded this as a very definite condition and it should not, in the writer's opinion, be classified among the phases of shock. The term "nervous shock" should be reserved for such phenomena as were comparable to the shock experienced in Goltz' experiment; namely, those observed in certain patients who, although they had suffered sudden and acute pain, had no bleeding wounds and in whom the symptoms characteristic of shock had rapidly appeared. Such cases were relatively rare.

2. *Infectious shock.* This class comprised cases in whom infection presented a particularly rapid and dangerous evolution. Since such patients were suffering only from infection the writer believed that the category should be abolished. He quoted two cases which had been cited before the French Surgical Society and which were described by one author as typical of nervous shock and by another as typical of septico-toxemic shock.

3. *Hemorrhagic shock.* Loss of blood was the principal factor in shock, and, with very rare exceptions, cases diagnosed as "shocked" had suffered from hemorrhage. Army surgeons appeared to agree that, with the exception of cerebral commotion cases and some abdominal wound cases, shock was not evident at the first dressing station. Numerous instances had been cited of severely injured men who were able to walk for a long time seeking shelter, and who were despatched towards the ambulance without presenting characteristic signs of shock. Some hours later the same cases would show symptoms of shock — imperceptible pulse, chill, etc. This was secondary shock. Between the time the wounded man left the dressing station and the time he arrived at a surgical center he was exposed to many adverse circumstances : repeated hemorrhages, pain, chills, sometimes long intervals of waiting and emotion. These conditions might be complicated by a tourniquet and, after a certain lapse of time, by the development of infection. In this way a man who was primarily suffering merely from hemorrhage and the effects of hemorrhage might, after some hours, be found suffering from shock with a very serious prognosis. It was, unfortunately, exceedingly difficult to estimate the amount of blood which might have been lost, and to decide whether there had been a hemorrhage or whether the case was exclusively one of nervous shock. It was only at autopsy, when, perhaps, lesions of an unsuspected extent were revealed, that the rôle which hemorrhage played among the shocked was realized.

Most authors who had treated the question had dwelt upon the characteristics common to both shock and hemorrhage, and they had all insisted upon the difficulty of making a differential diagnosis. Would it not be more logical to attach greater importance to the clinical resemblances instead of endeavoring at all costs to establish differential characteristics which must always remain very uncertain?

4. *Toxic shock.* The idea of attributing certain cases of shock to intoxication, the writer remarked, was a recent one; it had been suggested that re-absorption of fats, albuminous matter, etc., might take place at the site of the wound. This was possible, but, up to the present, a mere hypothesis. Advocates of this theory advanced, as proof, instances of rapid improvement by excision of the wound in certain cases of shock.

The writer stated that it was not his intention to attribute shock wholly to hemorrhage. He thought the part played by hemorrhage was considerable and that it was very often the origin of accidents which developed secondarily into shock; and he also thought that sufficient stress had not been laid upon these facts in the pathological history of the greater number of shock cases. Other causes certainly existed, however, capable in themselves, apart from all hemorrhage, of producing primitive shock, or of adding their action to that of the loss of blood. In wounds of the abdomen, undoubtedly, there was an additional factor which increased the rapidity of the process of shock. The rôle played by pain and emotion must also be taken into account in these cases.

*Treatment.* — The prognosis in cases of shock, the writer declared, was very difficult to establish. It was necessary to consider the extent and number of wounds, the time elapsed before reception of the patient, the effect of a tourniquet (as this might aggravate infection), and the existence of vascular lesions. The development generally became manifest, however, during the first hour of treatment and if, after that time, the tension had not been raised and maintained and the temperature had not risen, there was little hope of a favorable course.

Morphine and warming of the patient had given better results than any other method of treatment. The writer said he had used for heating a very simple apparatus consisting of a half cylinder and four lamps of 50 candle-power with carbon filaments which produced a temperature of $40^0$ in 5 minutes and of $60^0$ in 15 minutes. If electricity were lacking, a simple alcohol lamp in any kind of receptacle, placed at the bottom of the bed to introduce warm air under the coverings would quickly raise the temperature to about $50^0$. It was important never to use overheated operating rooms. Warming afforded evident relief to the patient, and, when there was no hemorrhage, helped to reestablish a normal distribution of the blood stream and draw the blood to the periphery.

In some cases the writer had made infusions of a *gummed and calcified serum*, prepared according to the formulae of Bayliss in England and of Delaunay in France. The mineral sera saline solutions and Locke's serum — were powerless in themselves to relieve the pressure in a wounded man who had been subjected to a severe hemorrhage. The addition of a colloid substance such as gum-arabic improved the composition in that the artificial serum thus obtained was from a physio-chemical point of view similar to that of the blood itself. Experimentally, reanimation after bleeding could be obtained by Locke's serum to which gumarabic had been added when saline solutions often proved of no avail. When hemorrhage was involved the injection of Locke's serum, gummed and calcified, excited the cardio-vascular system and thus produced an increase of pressure which was often permanent. The writer employed Locke's solution, to which gumarabic had been added in the ratio of 3 : 100, administered in the usual doses.

Taking into consideration the preponderant rôle attributable to hemorrhage in the majority of cases of shock, and also the fact that in such cases infection was particularly rapid and severe, the author had come to believe that the only effective therapeutic treatment for shock was *preventive treatment*. This method, however, involved the treatment of all patients with multiple wounds or with extensive shattering of the limbs, and those with tourniquets, in the forward surgical formations. Such formations were usually reserved for abdominal cases, but it was very doubtful whether, seeing the very delicate nature of the operations necessary, it was really advisable to perform them under the adverse conditions present in an advanced post during action. If shock cases were treated at these posts instead of the abdominal cases, amputations might be practised at an earlier moment — *and thus a great many cases of secondary shock with a fatal prognosis might be avoided.*

As regards the desirability of surgical intervention during the period of shock, the writer had observed that operations on the skull or on the chest never seemed to aggravate the condition; in abdominal cases the rapidity with which the shock developed indicated the presence of an important factor other than hemorrhage, and intervention under such conditions was rarely attended with success. For wounds of the extremities it was advisable, when a member was doomed, to amputate; as amputation might be performed rapidly under slight anesthesia and in such a manner that the patient might not lose a drop of blood. In the case of wounds which, under ordinary conditions, would not entail loss of a limb, the question was more difficult to decide, especially when there were multiple lesions. Excision of the wounds was contra-indicated on account of the length of the operation, the prolonged anesthesia necessary, and the inevitable loss of blood; in vascular lesions the vessels might be ligated; when a large artery had been manifestly injured it might be sutured, but when, after careful consideration, the surgeon believed that amputation would probably be necessary at a future date, it was much better to perform it immediately and rapidly. For men in a shocked or incipiently shocked condition, long operations should be postponed until there was an improvement in the general condition; the author had no illusions concerning the dangers of infection to which such delay exposed the patients, but he believed " waiting " treatment gave them the best chance.

The Subject of the Morning Meeting, March 16, was Hemorrhage and Transfusion.

Captain O. H. Robertson, M. R. C., read a paper on transfusion with preserved red blood cells. He called attention to the need of a method for giving transfusion rapidly. The difficulty of procuring blood for transfusion under the rush conditions near the front, makes this practice almost impossible in nearly all cases in which it is indicated. The time required for the transfusion is another disadvantage. The use of preserved human blood cells for transfusion is a possible solution of certain of these difficulties.

Rous and Turner[1] working at the Rockefeller Institute for Medical Research, found that blood taken into a solution of dextrose and sodium citrate and kept in the cold could be preserved for several weeks. Red blood cells thus treated remained intact and kept their viability for a long period. The preserving fluid used consisted of 5.4 o/o combined with 3.8 o/o sodium citrate; both of these solutions are isotonic with blood plasma. A mixture of three parts blood, two parts citrate, and five parts dextrose solution is placed in the ice-chest and the red cells allowed to settle. The supernatant fluid shows no signs of hemolysis. After washing in Locke's solution there is no apparent loss of integrity. Human red blood cells show no apparent deterioration at the end of four weeks. Experiments on rabbits indicated that red cells which have not deteriorated act as normal when transfused: if there is only slight deterioration without hemolysis, their effect when transfused is only temporary; if, however, there is any hemolysis, the effect produced is extremely harmful and the recipients die within twenty-four hours.

In experiments which the speaker was permitted to carry out at a casualty clearing station, the preserving solution was made up of 500 cc. of blood (the usual amount taken from a donor), 350 cc. of isotonic sodium citrate solution, and 850 cc. of " isodextrose ". These two solutions must be made up and autoclaved separately. Freshly distilled water should be used for all solutions. Powdered dextrose is preferable for the " isodextrose " solution, but liquid glucose which is more easily available has given satisfactory results. (For 850 cc. of a 5.4 o/o solution, 46 grammes of powdered dextrose or 57.5 grammes of liquid glucose is needed.) If the liquid glucose is used, it is found convenient to make up a large stock solution to keep on hand — about 360 grammes in a large enough amount of distilled water to bring the total volume of the solution to 1800 cc. It should be filtered and sterilized.

For making " isodextrose " 287 cc. of this concentrated solution is diluted with distilled water to 850 cc. and sterilized in the autoclave for half an hour at a temperature of 105° C. Above this temperature, the glucose is liable to decompose.

1. ROUS, PEYTON, and TURNER, *Jour. Exper. Med.*, Vol. XXII, p. 219, 1916.

Similarly it is convenient to make a 20 o/o stock solution of sodium citrate. This can be done by dissolving 90 grammes of sodium citrate in enough distilled water to raise the volume to 450 cc. It should be filtered and sterilized. For making "isocitrate" 66.5 cc. of the 20 o/o solution is diluted to 350 cc. with distilled water. Small bottles used for transfusion are each filled with this quantity and sterilized in the autoclave at a temperature of 105° to 110° C. for half an hour. Before sterilizing any of the solutions, the bottles should be plugged with cotton wool and covered with gauze.

The speaker described the methods used for securing and storing the blood, emphasizing the advantage of securing blood from donors belonging to blood group IV[1], because the blood cells of this group are not hemolyzed or agglutinated by the blood of any individual. Persons with a history of malaria, trench fever, or syphilis are excluded. The needle should be sharpened before each bleeding so as to offer a good spear point. If clotting occurs for any reason — such as slipping of the needle, or air leakage — and the blood ceases to flow, the tourniquet, applied to swell the veins, should be released, the stop-cock in the receiving tube closed, the needle withdrawn from the vein, and another attempt made with a fresh needle, rubber connection, and glass tube, using the same receiving bottle. It is better to take another vein. If the needle method fails, a cannula may be used, but this method is more difficult and attended with more danger of infection. Since varying amounts may be needed for transfusion, it is well to obtain 250 cc. quantities of blood as well.

The blood should be stored in a carefully constructed ice-box. The large bulk of the blood is slow in settling, so that it is not available for use until about the fourth or fifth day. If less blood is put into the same amount of fluid it settles somewhat more rapidly. A pink tint in the supernatant fluid should be regarded with suspicion, as it may signify hemolysis due to infection. This pink tint, however, may be due to the uneven settling of the red cells, in which case it will gradually disappear. At the end of the fourth week of keeping, disintegration of the cells is gradually indicated by a pink coloring of the supernatant fluid just above the cell layer. This appearance gradually extends towards the surface.

*Transfusion.* The supernatant fluid is drawn off by a syphon apparatus. In case the cells have sedimented to a volume less than

1. LEE, *Brit. Med. Journ.*, Nov. 24, 1917. For an abstract of this article see p. 458.

the original amount withdrawn, a diluting fluid, similar to Hogan's solution[1], may be used to restore the blood to the original quantity. Large fluid bulk is desirable in transfusion, so that the total volume transfused in each case is made up to 1000 cc. by the addition of the gelatin solution. Blood cells should not be poured when they are in a sediment more concentrated than normal blood.

The blood cell suspension should be gently agitated by a rotary motion and filtered through gauze into the transfusion bottle. The bottle should be tilted to allow the blood to flow down the side to prevent injury to the cells. The bottle when filled should be placed in a jar containing water at a temperature of from 106° to 107° F.

A small amount of novocaine is injected into the site chosen for the puncture. Care is exercised to prevent the admission of air into the vein. In case the patient's veins are in a state of collapse, it may be necessary to cut down on the vein and to use a cannula. Ten minutes should be the minimum time for the introduction of 500 cc. of blood. The amounts of blood given vary with the needs of the case, although the fluid amount is always made up to 1000 cc. by the gelatin solution.

By this method transfusion was practised 22 times on 20 individuals, most of whom were suffering from severe shock or hemorrhage, but with hope of recovery as a result of transfusion. In these cases the results were in all respects as favorable as might be expected from the transfusion of fresh blood. Hemoglobin estimations before and after transfusion and in some cases a red cell count were made. Hemoglobin estimations after preserved blood cell transfusion varied identically with those made after fresh blood transfusion. There were no reactions during the process or afterwards, and no evidence of increased blood destruction.

Of the 20 patients, 11 were discharged to the base hospitals in good condition. There were 9 deaths: one from profound anemia, and 8 from gas gangrene. Of this last group 3 cases showed marked improvement due to the transfusion before the appearance of the gas infection.

In meeting the objection that blood deprived of its plasma cannot

1. It may be prepared as follows: 25 gm. Gold Seal gelatin, 200 cc. distilled water, 2 gm. NaCL. The mixture should be boiled for 15 to 20 minutes in a water bath for the complete solution of the gelatin. While hot it should be filtered through a coarse filter paper and autoclaved for one hour at 124° C. When desired for use the gelatin is melted in warm water and poured into 800 cc. of sterile 0.9 o/o NaCL solution to which has been added 0.2 o/o Sodium Carbonate $NaCO_3 . 10 H_2O$. The water used should be freshly distilled.

be as efficient a restorative as whole blood, the speaker remarked that this was perhaps the case in conditions of low blood pressure due to shock, but that the gelatin solution, added to the blood cells, approximates the normal consistency of the blood. In simple cases of exsanguination, the red blood cells alone are quite sufficient to restore the circulation to its normal condition.

Among the advantages of transfusion by preserved blood, the speaker mentioned the possibility of having large quantities on hand in times of great activity, the time saved in giving the transfusion, the convenience of administering the blood with the patient in bed and without the presence of the donor, the possibility of giving large amounts of blood at a time, and the ease of transporting the preserved blood without injury for considerable distances.

Major Hamilton Drummond, R. A. M. C., gave the results of observations made on shocked cases covering a period of nine months in an advanced casualty clearing station about five miles behind the lines. Only the severely wounded were admitted, and the wounds were chiefly those of the abdomen, chest, and lacerated limbs.

The hospital was seldom crowded, so that the cases could be observed and studied to the end. Upon arrival, the patient was placed on an artificially heated bed and given hot drinks. The wounds were then examined and the blood pressure, pulse, respiration, and temperature noted. In most cases, if time permitted, a complete blood count and hemoglobin percentage was obtained by Captain Stuart Taylor. Cases arrived from 3 to 6 hours after wounding, having traveled from 5 to 10 miles.

The cases could roughly be divided into those due to loss of blood and those due to other causes, such as multiple wounds, severe bone or joint lesions, or hollow visceral injuries, all of which were frequently aggravated by cold and fatigue. Men blown up in a dug-out explosion, with very slight or no external injuries, often presented signs of profound shock similar to cases with severe anatomical damage. It was not always easy to distinguish cases due to loss of blood from those due to other causes, for often both types might occur in the same individual. It was most important to distinguish when possible.

It was often found that a pallid man with a low blood pressure and rapid pulse, was not necessarily a case for the transfusion of gum or blood. In making this distinction, the blood count was found to be of great importance. Captain Taylor, working on the lines of Cannon, Hooper and others, found the concentration in the capillaries in shocked cases. Working on the lines of Goeverts, he observed that a low red count in the early stages suggested severe hemorrhage and was an indication that transfusion of blood or gum was urgently needed.

The cases were thus divided into four groups :

1. Cases in which hemorrhage was severe and shock minimal.
2. Cases in which hemorrhage and shock were present to a moderate degree.
3. Cases in which hemorrhage was slight and shock very marked.
4. Cases that arrived at the hospital in a moribund condition.

Gum transfusion was extensively used in this series of cases (80) as donors for blood transfusion were not available. The 5 o/o solution used was prepared according to Bayliss' formula :

| | | |
|---|---|---|
| Sodium chloride. . . . . . . . . . . . . | 2 | grammes. |
| Pot. chloride. . . . . . . . . . . . . . . | 0.5 | " |
| Calcium chloride . . . . . . . . . . . | 0.5 | " |
| Powdered gumacacia. . . . . . . . . . | 5 | " |
| Distilled water. . . . . . . . . . . . . . | 100 | cc. |

*Group* 1. When hemorrhage alone was the cause, the injuries consisted chiefly of perforations of large arteries or veins, or of lesions in the solid organs, such as the spleen, liver, or kidneys. In such cases blood transfusion gave the best results, for the blood pressure was thereby considerably increased, and the pulse rate lowered to a marked degree. These results were maintained for a much longer period than when saline or hypertonic salt solutions were administered.

*Group* 2. When hemorrhage and shock were both present to a moderate degree, the results obtained by transfusion were fairly good. The transfusions were given either before or during an operation to prevent the fall of blood pressure to a dangerous level. The transfusion of gum was especially serviceable in that type of case in which an operation for multiple wounds extended over a considerable period of time. The rise in blood pressure was, however, less marked than in the previous group, in which hemorrhage alone was the responsible cause of shock.

*Group* 3. Cases suffering from marked traumatic shock, especially those resulting from much hollow visceral damage, showed little improvement as a result of transfusion. Experience showed that cases with severe abdominal wounds involving the liver were not benefited by transfusion, because the rise in blood pressure often caused the hemorrhage to recur, bringing on a fatal collapse.

The speaker stated that at present when transfusion is practised in cases of abdominal injury, it is not performed until the patient is under anesthetics. A surgeon is always present prepared to control immediately the bleeding points.

Similarly compound fractures and severe limb wounds may be endangered by a sudden rise in blood pressure. It has therefore been found advisable in such cases to apply a tourniquet before performing the transfusion. The speaker, to illustrate this point, cited the case of a man both of whose legs were severely shattered. The right leg was blown off in the middle third. The left leg had a compound fracture of the tibia and fibula in the middle third, and in addition, mutilation of the ankle joint and tarsus. The hemorrhage was still active when the patient arrived, 2 1/2 hours after injury. At that time the pulse was 95; temperature below 95° F.; respirations 22; B. P. systolic 76 mm. A tourniquet was immediately applied and transfusion with gumacacia was performed. At the end of the transfusion the blood pressure had risen to 122 mm., and the general condition had greatly improved. A double amputation was then performed, after which the B. P. fell to 92 mm. Twelve hours later it had risen to 104. The pulse rate was then 120. The patient made good recovery and was sent to the base 11 days later.

The technique for gum acacia transfusion is the same as that for normal saline. The gum is kept in pint bottles, being first filtered through paper pulp under pressure before it is autoclaved. The fluid should be introduced gradually into the circulation; from 15 to 20 minutes should be allowed for each pint. During the transfusion the solution is maintained at a temperature of 118° F.; a pint is administered at a time.

No ill effects of the introduction of the fluid into the circulation have ever been observed. At autopsies on cases in which death resulted from severe injuries, no pathological lesions due to the fluid (thrombosis, etc.) were found.

A report by members of the *Lakeside Unit* was presented.

It stated that since the unit arrived in France 320 transfusions of blood had been performed by the surgical staff of General Hospital N° 91 (*Lakeside*).

Indications for transfusion at the front are: (1) hemorrhage — either severe primary or early recurrent; (2) shock; (3) shock and hemorrhage. Pure shock and pure hemorrhage are rare occurrences; but the combination of the two are the rule.

Although diagnosis of the two conditions is often difficult, the following symptoms are fairly reliable as a means of differentiating. If hemorrhage is the cause of the collapse, the patient is usually pale and his lips blanched; he is restless, thirsty, and anxious. The shocked patient, on the contrary, has a grayish blue coloration. A more reliable symptom is the increase in the white cell count attending cases of hemorrhage but absent in shock.

At the base hospital, indications for transfusion are: (1) hemorrhage—severe, recurrent, secondary, or post-operative; (2) anemia, secondary to sepsis. In addition to the usual symptoms in this form of anemia, the red and white cell counts are of value and a hemoglobin of 30 or below appears to be a determining factor.

*Donors* must be free from acute infections—especially syphilis and tuberculosis. They are chosen from the lightly wounded or dental cases. Best results have been obtained when the recipient and donor are of the same blood group, but Group IV blood (Lee's classification) has been transfused successfully into recipients of the other groups.

All of the 104 instances of grouping were done with sera of Groups II and III preserved with 0.5 o/o carbolic, put up in sealed glass tubes by Captain Karsner. Since serum loses its power to agglutinate in a month or more, surgical teams are now made up with a man known to be of Group II and one of Group III among its members so that a fresh supply may be had readily when needed.

When donor and recipient were of the same group, no incompatibilities were observed. One unfavorable reaction occurred when a Group IV (universal) donor was used on a recipient belonging to another group.

Unmatched blood was used in 216 cases, in the majority of which no signs of incompatibility were observed. In 51 of these cases chilly sensations but no distinct rigors were reported. In no case was there hemolysis, although the urines were not examined for hemoglobin. In one case there was a severe reaction resulting in death. In another case a sharp reaction was observed during transfusion, but the transfusion was stopped after 300 cc. had been given, and no further difficulty occurred. In another instance a slight reaction occurred after each of two transfusions, the last following loss of blood by secondary hemorrhage after amputation. On the third day after the second transfusion the patient had a slight rash like that from serum injections, and a temperature of 101° F.

Unfavorable symptoms attributable to blood incompatibility to be looked for are: precordial pain, increase in the pulse rate, failure to improve in color, heart distension, marked unrest, and widely dilated pupils.

Various methods of transfusion were employed. In most cases paraffin-lined tubes were used. In 2 cases Linderman's syringe was used. In 2 cases, owing to an absence of the proper apparatus, blood was collected into an equal volume of 2 1/2 o/o saline and given as an ordinary intravenous injection with good results. Harrison used blood preserved according to Robertson's method, but owing to the difficulty of preparation, he prefers fresh whole blood.

The results from treatment were good in cases of anemia due chiefly to hemorrhage, and in a certain number of cases in which hemorrhage was accompanied by shock; also in some cases of sepsis and anemia in which the anemia was the chief factor. In cases of anemia from hemorrhage accompanied by profound shock, from wounds beyond repair and in cases of anemia and sepsis in which sepsis was the principal factor, blood transfusion was of little avail.

A paper entitled " A Note on Hemorrhages " by Doctor C. JANSSEN, of the Belgian Medical Corps, was read in translation. The writer on behalf of the Belgian physicians expressed gratitude for the sympathy shown by their American colleagues during the great national trial.

The writer remarked that war wounds at present are more frequently accompanied hy hemorrhage than was formerly the case, and he attributes this fact to battle conditions and to the nature of the projectiles. Wounds from shells and bombs are becoming more frequent than those from balls, and they are more likely to result in serious hemorrhage because of the size and irregularity of their entrance and exit orifices, and also because of the destructive nature of their action upon the internal anatomy.

Hemorrhage in wounds with small openings is less likely to be serious than in wounds with large openings, because, even if important vessels are cut, the external loss of blood is less. Hematoma thus formed, however, may, by exerting pressure, impede the collateral blood supply, and so favor ischemia and gangrene. If the arterial wound is only lateral, a diffuse aneurism may develop, but the prognosis is less serious.

In the case of wounds with large openings, on the other hand, hemorrhage is greatly to be feared. In such wounds complete section of an artery is less serious vitally than a lateral wound of the vessel, because an artery completely cut by a shell fragment is likely to be constricted in such a manner as greatly to reduce the flow of blood under conditions which generally favor the formation of clots. In large open wounds exposing a lateral injury of an artery, however, hemorrhage is generally fatal.

Hemorrhage is often prevented by an early formation of a clot or by a fragment of missile which obstructs the passage of blood. In transportation the clot thus formed is sometimes dislodged and a serious hemorrhage brought about. Similarly when a missile is removed on the operating table, a hemorrhage may result in such a manner as to make the isolation and ligature of the vessels extremely difficult. For this reason it is better to delay the removal of missiles until all the vessels have been sought out and temporarily ligatured.

Internal hemorrhages in the limbs are not generally fatal, but if a hemorrhage issues into a body cavity, the prognosis is most serious. In wounds of the lung, hemorrhage is especially dangerous. Out of 320 such cases, there were 36 deaths within the first 24 hours. Intraperitoneal hemorrhage is also dangerous, especially if any large trunk is injured. Wounds of the spleen, liver, and of mesenteric vessels are chiefly to be feared. It is often difficult to tell precisely the source of a hemorrhage resulting from an abdominal wound. Doctor Govaerts has observed that internal hemorrhages proceeding from abdominal organs show an increase in erythrocytes and leucocytes. The increase in red cells, however, is only temporary, continuing but a few hours after the injury.

Internal cerebral hemorrhages are generally serious. Tachycardia and the Cheyne-Stokes' respiratory rythm combined indicate a hemorrhage with the prognosis of early death. Sub-dural hemorrhages are not common in open wounds of the brain. The most serious hemorrhages from brain injuries are those resulting from wounds of the sinuses of the dura mater. Occasionally a bone fragment or a missile produces hemostasis, and hence in operating, fragments should be removed last.

*Treatment.* At the *poste de secours*, a garrot may be applied when there is a formidable hemorrhage due to an injury of a principal artery. Otherwise a thick tight bandage serves to stem the blood flow, and is preferable to a garrot because it is less likely to bring about a stoppage of the circulation which might favor the development of gas gangrene. All [such cases should be hurried to an operating station. From a mortality table setting forth the results from 92 cases of vascular injury, the writer concludes that ligature of important vessels is dangerous, not so much because of ischemic gangrene as because of gas gangrene.

M. Janssen advises a sparing use of ligatures. They should be applied only in case the vessel is completely severed or excessively damaged. The corresponding vein should also be ligatured. In case of complete section of vessels together with serious bone destruction or devitalization of muscle inviting gas gangrene, one need not hesitate to amputate. If amputation is delayed, a careful watch should be kept against gaseous infection.

Vessel suture is rarely indicated and very seldom successful. It may be accomplished in case a large vessel is only partially severed. The writer has never had any success from inserting a paraffined tube.

Internal hemorrhages in the limbs may be treated by removing the clot and by ligature; but if the hematoma is not serious, it is well to delay operation to insure the reestablishment of the circulation. Hemorrhages in the peritoneal cavity must be treated by immediate laparotomy. Wounds in the liver may be plugged. Splenectomy may often be practised. Suture is possible in wounds of the kidneys. Ligature of the mesenteric vessels often calls for intestinal resection, which is always more serious than simple intestinal suture.

The writer outlined the practice of the "Ambulance de l'Océan" in cases of thoracic hemorrhage as follows : The patient is immobilized on an electric bed, and given an injection of morphine. The blood pressure is taken every half hour and a blood count made. If it is clear that the hemorrhage persists, thoracotomy is practised, with local anesthesia when practicable, and as quickly as possible. A few catgut stitches suffice to close the parenchyma and to insure hemostasis. If a bleeding vessel is observed, it should be ligatured immediately. After the pleura is dried the cavity should be closed without drainage.

In brain wounds, ligature is sometimes possible, but in most cases in which a sinus is involved, plugging is necessary. Hemorrhages deep in the tissue defy intervention.

Secondary hemorrhage is no longer as frequent as at the beginning of the war, because surgical technique has improved, and because more radical treatment is given at the start. Plugging of a wound for hemorrhage should be avoided entirely except as a late measure.

A state of shock due primarily to hemorrhage is considered as having a dangerous prognosis [if the red cell count falls below 4,000,000 per cc. Intravenous injections of Ringer's solution and hypodermic injections of other products, have only a temporary effect. Depage and Govaerts consider transfusion of blood to be essential in all cases in which the red cell count falls below 4,000,000 within 6 hours after wounding.

Blood transfusion has given remarkable results. The speaker declared that transfusion could be effected successfully and easily by ordinary syringes. For a transfusion some ten glass syringes with a contents of 20 cc. are necessary. One person draws the blood, another injects it, and the third rinses the syringes. The blood remains in the syringe only about 20 seconds, and is injected before coagulation takes place. Transfusion practised in this fashion is very simple and requires no special apparatus.

By transfusion 8 out of 14 cases with a red cell count of 4,000,000 or less per cc. were saved whereas out of 14 similar cases in which transfusion was not performed, 13 died.

The author believes that blood transfusion in cases of serious hemorrhage has been amply justified by the results, and he urges a wider use of this efficacious form of treatment.

The Subject of the Afternoon Meeting, March 16, was Anesthesia.

Dr. Desplas read a paper on spinal anesthesia by means of Stovain, which he had found particularly efficacious in war surgery, as it permitted patients suffering from shock to be operated with more hope of recovery than when general anesthesia was employed. It was indicated for operations on the lower extremities, abdomen, and lumbar region. The speaker based his remarks on 571 cases which he had personally operated.

### TECHNIQUE

As a preliminary the patient should be told what to expect, and each detail of the procedure should subsequently be explained to him in advance. To lessen psychic control 1 centigramme of morphia should be injected half an hour beforehand. The whole of the lumbar region should be painted with iodine.

Materials required (all carefully sterilized) : — one 2 cc. Luër syringe; one 4 cc. aspirating needle; one Tuffier steel lumbar puncture needle 7.5 cm. long (Collin), ampoules of 10 o/o Billon Stovain, each containing 0.75 cc. (i. e. 7.5 centigr.) of Stovain in a solution of sodium chloride.

1. *Seated Position.* Patient should be placed at edge of table, feet resting on a stool, arms crossed, back maintained in a bent position by the help of an orderly. The puncture should be made in the space between the second and third vertebrae instead of between the third and fourth as in the classical manner, for the high puncture enables an anesthesia to be obtained which reaches the base of the thorax and of which the quality and constancy are superior to those of the anesthesia obtained by low puncture. The index finger of the left hand seeks the space between the 2nd and 3rd vertebrae by the inferior spinous process at 1 cm. above the latter, in the centre of the inter-spinous space, on the median line; the Tuffier needle (which must contain its stilet) is inserted perpendicularly in the dorsal plane. At a depth of from 4 to 6 1/2 cm , varying with the subject, the sensation of " piercing " the wall of a cavity is felt; this indicates that the needle has penetrated the dura-mater. The stilet is withdrawn, and 1 cc. of spinal fluid is allowed to exude.

The Luër syringe, containing 1/2 cc. of solution, is then placed on the pavilion and the operator aspirates slowly; the syringe fills and the spinal fluid dilutes the stovain solution. An opalescent cloudiness is rapidly produced, and as soon as it becomes unmistakable the content of the syringe is pushed into the dural cavity. The operator *aspires slowly*, pushes *very* slowly, draws the needle and syringe out sharply, and the patient is slowly laid down; he is placed in a horizontal position with the head considerably raised.

All these operations must be performed in complete silence, with extreme gentleness, and as slowly as possible. It has been proved that hurried injections irritate the spinal cord and provoke a lowering of the arterial pressure.

2. *Lateral Decubital Position.* For cases where considerable traumatism is present and in which the shocked condition is very marked. An orderly supports the neck of the patient with his right hand, with his left seizes the posterior surface of the knee joint, and, approaching his two hands, presents the patient's back to the operator in such a position that its lumbar plane is perpendicular to the table. The remainder of the technique is the same, but in this position it is better not to make the puncture on the median line but at 1 cm. beyond, on the lateral edge of the inter-spinous ligament, the needle following the lateral edge.

The stovain must never be injected until the flow of spinal fluid has been seen.

Anesthesia is complete in from six to seven minutes and lasts from fifty minutes to one hour and a half. If sensation has not been completely inhibited the injection has been too hurried or the surgical operation has begun too soon.

The usual dose is 1/2 cc. of a 10 o/o solution, i. e. 5 centigr., but for patients who have been operated several times at short intervals it is necessary to increase it to 6 or 7 centigr.

Absolute silence must be preserved in the operating room throughout the subsequent proceedings.

Should the patient show signs of discomfort, a hot drink should be given or ether inhaled.

After the operation the patient should be laid flat in his bed and not allowed to speak. During the first few hours he may feel a sensation of heat and of pricking in the lower extremities. He may drink four hours after operation and begin to eat the following day.

Dr. Desplas had never observed syncope, vertiginous phenomena, motor troubles of the lower extremities, sphincterial weaknesses, or disturbances of the equilibrium. In 5 cases headaches were cured by aspirine and in 2 cases by lumbar puncture to relieve pressure caused by an abnormal amount of spinal fluid. Out of 571 cases no post-operative death was due to the spinal anesthesia. Retention of urine, observed in a few abnormal cases and due rather to the handling of the cord than to the anesthesia, is not a matter of any importance provided asepsis and the evacuation of the bladder are practised.

*Operative Advantages of Spinal Anesthesia.*

1. No special anesthetist is needed.

2. Under spinal anesthesia any special treatment necessitated by the shocked condition of the patient may be easily given.

3. The possibility of pulmonary, renal, or hepatic complications, such as result from general anesthesia, are excluded.

4. There is no post-operative vomiting.

5. By spinal anesthesia complete relaxation of the muscles is obtained as under no other anesthesia. This condition is especially helpful in :

*a*) Extensive laparotomies, as the intestinal mass has no tendency to protrude and shock due to malaxation is thus, *ipso facto*, almost non-existant.

*b*) Operations for extensive shattering of the lower members.

The speaker added that patients who have experienced both methods prefer spinal anesthesia.

Experiments were undertaken which proved *a*) that the stovain cannot cause disturbances in the region of the nerves of the *cauda equina* because it is eliminated entirely and rapidly in the urine; and *b*) that the reputation of spinal anesthesia for causing shock is unjustified. These experiments extended over forty patients in each case.

*a*) Proof that stovain could not cause disturbances in the region of the nerves of the *cauda equina* :

1. The opalescent cloud produced in the syringe by the mixture of the stovain solution and the spinal fluid is due to the action of the bicarbonate of soda contained in the spinal fluid on the hydrochloride of amylene which constitutes the stovain. It is a phenomenon of the saturation of an acid by a base.

2. Stovain being an alkaloid is eliminated through the urine; for the non-albuminous urine of an anesthetized patient, treated by Tanret's reagent, gives a precipitate which is insoluble in alcohol (the characteristic reaction of alkaloids).

3. Under experimental conditions the elimination of the stovain through the urine is complete in 9 hours — the average is between 6 1/2 and 8 hours.

The eliminatory process is as follows :

| | | | |
|---|---|---|---|
| After | 2 hours | elimination of | 3 centigrammes |
| " | 4 " | " | 1 " |
| " | 6 " | " | 1/2 " |
| " | 8 " | " | a quantity too minute to calculate. |

*b*) Proof that the reputation of stovain for causing shock is unjustifiable :

The arterial pressure of patients was taken, by means of Pachon's sphygmomanometric oscillometer, before the intraspinal injection of stovain, every five minutes during the course of the operation, and every 4 hours during the 24 following it. Among the 40 cases observed many were already in a condition of shock.

1. In 35 cases the arterial pressure present before the anesthesia was maintained.

2. In 3 cases there was a slight lowering of the arterial pressure — down to 20 mm. below the minimum pressure.

3. Spinal anesthesia could not be considered responsible in the 2 very serious cases in which the process of shock continued to evolve in spite of all cardiac stimulants.

4. A considerable fall in arterial pressure takes place in patients operated under general anesthesia.

*Results.*

| | Number of operations | Post-operative deaths |
|---|---|---|
| Lower extremities and pelvis : | | |
| Amputations for crushed legs and gas gangrene | 27 | 4 |
| Sequestrectomies | 40 | 3 |
| Arthrotomies for removing projectiles lodged in articulations | 10 | |
| Resections of joints, primary and secondary (hip, knee, and tibio-tarsal) | 13 | 1 |
| Bone and osteo-synthetical sutures | 10 | |
| Extensive wounds of the lower limbs with or without vascular lesions | 89 | 3 |
| Extraction of projectiles | 125 | |
| Laminectomies for projectiles lodged in marrow cavity | 1 | |
| Plastic operations | 87 | |
| Luxations of the hip | 2 | |
| Fixing of apparatus | 6 | |
| Abdomen : | | |
| Laparotomies for penetrating wounds | 14 | 7 |
| Iliac anus | 1 | |
| Para-peritoneal wounds | 4 | |
| Traumatic rupture of the urethra | 1 | |
| | 430 | 18 |

| | Number of operations | Post-operative deaths |
|---|---|---|
| General surgery : | | |
| Cholecystectomy and drainage of the bile duct | 1 | |
| Inguinal and crural hernias | 94 | |
| Varicose veins | 8 | |
| Hydroceles and hematoceles | 5 | |
| Acute and gangrenous appendicitis (systematic appendectomies) | 15 | |
| Chronic appendicitis | 6 | |
| Hemorrhoids (Whitehead) | 12 | |
| | 141 | |

The total mortality was thus 4.18 per cent for war surgery and 0 per cent for general surgery. Of the former, 10 cases suffering from extensive abdominal wounds, shattered limbs, enormous fractures, or great loss of blood from various injuries to the vascular system, succumbed some hours after operation. Eliminating these, which were practically hopeless cases, the post-operative mortality percentage was 1.90.

Spinal anesthesia was easy, rapid, certain, economical, and safe.

Administered by the surgeon himself it produced no complications, and, since it did not increase shock, it enabled severely shocked patients (whose condition precluded the use of any general anesthesia) to be operated. It facilitated operative technique by affording complete relaxation of the muscles. It was, however, necessary to carry out rigorously its very simple technique. As much care must be exercised as for general anesthesia, and no detail, however trivial, must be neglected, as each possessed a certain physiological importance which must on no account be disregarded.

Captain GWATHMEY, M. R. C., demonstrated an apparatus for administering oxygen gas separately or in combination with ether. He stated that he had taken up the study of this problem with Dr. Woolsey of Brooklyn eighteen years ago, and had come to the conclusion that a " side-feed " apparatus which could be so constructed as to weigh only 5 or 10 pounds, was the least complicated and the most practical device. In France, the committee dealing with this problem, had chosen, in connection with the side-feed apparatus, the large cylinders (such as the speaker exhibited) because they required only one handling.

An apparatus for the actual administering of the anesthetics had been contrived by the speaker working with others. This appliance did away with the big reducing valves and clock dials, both of which were likely to get out of order. The percentage of nitrous oxide and oxygen was clearly indicated by the bubbles passing through the visible water receptacle. When ether was needed it could quickly and easily be added by turning another valve. The appliance was so simple that nothing could be taken away from it, although many things might be added. The total cost was about 100 francs.

The speaker said that he had been fortunate in having an opportunity to work with Captain Marshall, which experience had convinced him of the superiority of nitrous oxide and oxygen in military surgery.

In considering the subject of anesthetics it was necessary to distinguish between the needs in the army zone and those further back in the clearing station and the base hospital. Morphine, which alone was used in the ambulance service, if given in large quantities, caused the formation of lactic acid, hence the necessity of an additional analgesic. The difficulty of performing painful dressings had led the speaker to take up the study of oral analgesia. Hundreds of thousands of operations had been performed with gas and oxygen with the patient perfectly conscious, but with the pain pain slightly lessened. An ideal analgesia is now available by the combination of morphine and oral analgesia in the advanced zone, and of oral analgesia, morphine, and $N_2O-O_2$ in the other zones.

The speaker then took up the subject of general analgesia by oral administration. He stated that what he had to say was by way of a preliminary communication based on animal experiments and upon a sufficient number of clinical cases to support the conclusions from the experiments. It had been shown that general analgesia with loss of sensation, but without loss of consciousness, could be produced for painful dressings and for short operations. Although such a method had its advantages in civil surgery, it was doubly important in military hospitals.

The use of analgesia was especially helpful in dealing with cases of bone fracture. In such cases, it was highly important to keep the patient quiet. Since, however, the first few dressings were so painful as to require light anesthesia, the patient usually had to be removed to the operating room for the dressing. The technique evolved as the result of the study undertaken by the speaker, had made it possible to produce analgesia by oral administration with perfect safety, and without moving the patient from his bed.

This method was applicable to all forms of painful dressings, and was also being developed to apply to short surgical operations, such as the resection of a rib, the removal of foreign bodies, and revision of wounds. In such operations, analgesia might be supplemented by the use of novocain or the injection of morphine or by inhalation of anesthetics, or a combination of these methods.

In experiments upon rabbits, the following substances were administered by a stomach tube : quinine and urea hydrochloride (nikalgin); trional (dissolved in alcohol, 1 gr. to 1.5 cc.); morphine tartrate (in water); paraldehyde; ether (in olive oil — 50 o/o); and ether (in olive oil — 25 o/o); paraldehyde with 25 o/o ether in olive oil added; and albolene in various combinations with morphine tartrate, ether, and paraldehyde.

In the rabbits ether in oil proved the most successful analgesic by oral administration, but it produced acute gastritis. It was later discovered that olive oil alone produced justae severe gastritis. It was thought safe to try this combination in man, since both ingredients were known to be practically harmless in the human stomach. To make sure that no irritation should result from the menstruum, liquid paraffin (albolene) or Russian mineral oil were used instead of olive oil.

Clinically the following combinations were tried :

| | | |
|---|---|---|
| 1. | Ether. . . . . . . . . . . . . . . . . . . . | |
| | Liquid paraffin. . . . . . . . . . . . . . . . | āā 20 cc. |
| | Aq. menth. pep. . . . . . . . . . . . . . | 5 drops. |
| 2. | Paraldehyde. . . . . . . . . . . . . . . . | 5 to 15 cc. |
| | 50 o/o ether in Albolene . . . . . . . . . | 20 cc. |
| | Aq. menth. pep . . . . . . . . . . . . . | 5 drops. |

The objectionable taste of the mixtures with paraldehyde, and, to a less extent, with ether, was overcome by having the patient hold in his mouth a swallow of port wine, for about 30 seconds before taking the analgesic, which is followed immediately by another swallow of the wine. It was found that paraldehyde served no useful purpose, so that all the dressings were done with formula 1. Although it was well not to give the analgesic right after a meal, no special preparation of the stomach was necessary.

The speaker outlined 14 cases (selected from several hundred) in which oral analgesia was tried with wholly satisfactory results. They consisted of painful dressings, performed under analgesia without removing the patients from the wards. In some instances the patient was able to talk but unable to feel the pain of the dressing. In many instances the patient slept through the dressing, and although there was an occasional groan, no patient had any recollection of pain upon regaining consciousness. In cases, of extremely painful dressings during which the patient retained consciousness and had a sense of pain, the pain was much less than normal. In only three instances were the results reported other than excellent, and in one of these, the dressing was performed too soon after the mixture had been taken, and, in another, only a half dose was administered. In only one case did nausea result from the analgesic.

This method has also been used effectively in the service of Captain D. C. Taylor, R. A. M. C., in a casualty clearing station. The substitution of chloroform for paraldehyde in the following formula has given good results: it has been used in 30 cases :

| | |
|---|---|
| Chloroform. . . . . . . . . . . . . . . . . | 5 to 10 cc. |
| Ether. . . . . . . . . . . . . . . . . . . . | |
| Liquid paraffin . . . . . . . . . . . . . . . | āā 20 cc. |

The speaker stated that no detailed study had been made of the physiology of general analgesia by oral administration. Some of the advantages of the mixtures recommended were that oil and ether mixed perfectly and that the rate of absorption remained constant, so that the patient did not receive an overdose at one time and an insufficiency at another. The rate of absorption by the stomach was quicker than by the colon. For operative purposes it was necessary to supplement the analgesic by some other means, such as local anesthesia or inhalation. The amount that could be safely administered orally was not yet determined, but there could be no question that so long as a state of general anesthesia was not produced, there could be no danger from the amount of the mixture necessary to produce analgesia. If this method were adopted it would release a large number of anesthetists for other medical work[1].

NOTE. — The text of addresses by Captains Moss and Marshall was not received in time to be inserted in this number. They will be reported in the next issue of the *Bulletin*. Captain Burton J. Lee's demonstration of blood transfusion appliances is omitted because the report of the Committee on Blood Transfusion is to be published in pamphlet form.

1. For details of the experiments and clinical experience, see B. M. J., March 2, 1918.
2. See p. 458.

